# Molecular hydrogen protects against oxidative stress-induced SH-SY5Y neuroblastoma cell death through the process of mitohormesis

**DOI:** 10.1371/journal.pone.0176992

**Published:** 2017-05-03

**Authors:** Yayoi Murakami, Masafumi Ito, Ikuroh Ohsawa

**Affiliations:** 1 Biological Process of Aging, Tokyo Metropolitan Institute of Gerontology, Tokyo, Japan; 2 Research Team for Mechanism of Aging, Tokyo Metropolitan Institute of Gerontology, Tokyo, Japan; Massachusetts General Hospital/Harvard Medical School, UNITED STATES

## Abstract

Inhalation of molecular hydrogen (H_2_) gas ameliorates oxidative stress-induced acute injuries in the brain. Consumption of water nearly saturated with H_2_ also prevents chronic neurodegenerative diseases including Parkinson’s disease in animal and clinical studies. However, the molecular mechanisms underlying the remarkable effect of a small amount of H_2_ remain unclear. Here, we investigated the effect of H_2_ on mitochondria in cultured human neuroblastoma SH-SY5Y cells. H_2_ increased the mitochondrial membrane potential and the cellular ATP level, which were accompanied by a decrease in the reduced glutathione level and an increase in the superoxide level. Pretreatment with H_2_ suppressed H_2_O_2_-induced cell death, whereas post-treatment did not. Increases in the expression of anti-oxidative enzymes underlying the Nrf2 pathway in H_2_-treated cells indicated that mild stress caused by H_2_ induced increased resistance to exacerbated oxidative stress. We propose that H_2_ functions both as a radical scavenger and a mitohormetic effector against oxidative stress in cells.

## Introduction

Administration of molecular hydrogen (H_2_) has the potential to improve many diseases such as ischemic brain and heart infarctions, glaucoma, stress-induced cognitive decline, Parkinson’s disease, metabolic syndrome, and inflammatory diseases [1–3]. As a weak reductant, H_2_ rapidly diffuses into every tissue and cell and selectively scavenges highly toxic reactive oxygen species (ROS) including the hydroxyl radical (·OH) and peroxynitrite [4]. H_2_ can be administered or taken into the body by numerous routes. These are roughly classified into three types: inhalation of H_2_ gas, drinking of H_2_-dissolved water (HW), and injection of H_2_-dissolved saline. Administration of H_2_ varies depending on the disease. However, when HW is orally administrated, the amount of H_2_ is too small to detoxify a huge amount of ROS in the diseased tissue. The molecular mechanisms underlying the remarkable effects of a small amount of H_2_ remain unclear.

Several lines of evidence indicate that ·OH generated by ionizing irradiation of water reacts directly with H_2_. We also confirmed that the dissolved H_2_ reduces ·OH produced by the Fenton reaction, ultraviolet irradiation, or sonication *in vitro*, as judged by the fluorescence of 3'-*p*-(hydroxyphenyl) fluorescein and the electron spin resonance spectrum of 5,5-dimethyl-1-pyrroline *N*-oxide [4–6]. The reduction of ·OH by H_2_ was further observed in cultured cells, male germ cells in mouse, and retinas in rat [4, 7, 8]. These observations indicate that a sufficient amount of H_2_ can efficiently moderate oxidative damage induced by ·OH. On the other hand, H_2_ indirectly reduces oxidative stress by inducing anti-oxidation systems *in vivo*. Treatment with H_2_ induces hemeoxygenase-1 (HO-1), superoxide dismutase (SOD), and catalase, and reduces cyclooxygenase-2 and endothelin-1 (ET-1) expression [9–12]. In Nrf2-deficient mice, the therapeutic effects of inhaling H_2_ gas on hyperoxic lung injury decline with decreasing expression of HO-1 [13], indicating that activation of Nrf2 is involved in the biological pathways underlying the effects of H_2_.

In the current study, we investigated the effect of H_2_ on mitochondria in cultured neuroblastoma SH-SY5Y cells and found that H_2_ increased the mitochondrial membrane potential (ΔΨ_m_) and cellular ATP level, with an accompanying decrease in the reduced glutathione (GSH) level. Pretreatment of cells with H_2_ suppressed H_2_O_2_-induced oxidative stress, whereas post-treatment did not. These results raise the possibility that H_2_ functions differentially as an inducer of an adaptive response and as a radical scavenger in cells.

## Methods

### Cell culture and H_2_ treatment

Human neuroblastoma SH-SY5Y cells (ATCC CRL-2266) were maintained in Dulbecco’s modified eagle medium (DMEM) containing 10% fetal bovine serum (FBS), 25 mM HEPES, 1 mM pyruvate, penicillin-streptomycin, and 10 mM glucose (Glc) or galactose (Gal). Culture with Gal as the sole source of sugar forces mammalian cells to rely on mitochondrial oxidative phosphorylation (OXPHOS) [14].

**Fig 1:**
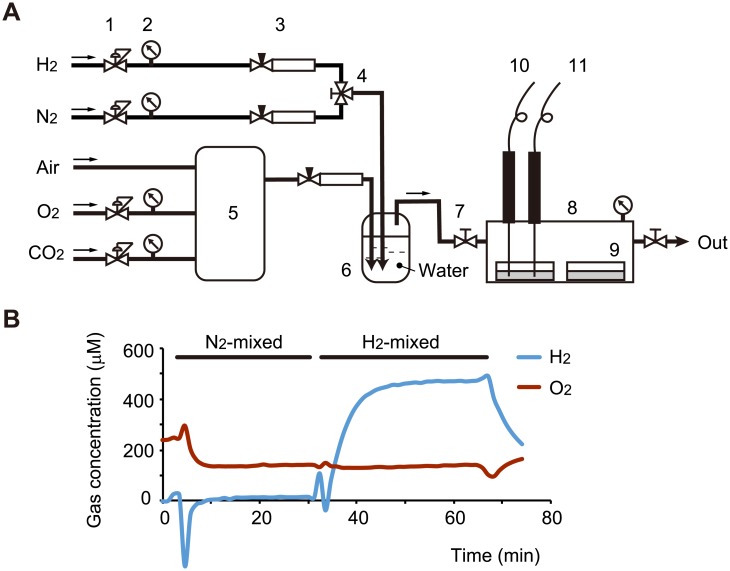
Cell culture system under an atmosphere containing H_2_ gas. (A) Schematic representation of the culture system. 1. Pressure-reducing regulator. 2. Pressure gauge. 3. Flowmeter with flow control valve. 4. Three-way plug valve. 5. Multi-gas controller. 6. Bubbler bottle. 7. Gate valve. 8: Acrylamide box. 9. Culture dish. 10. H_2_ electrode. 11. O_2_ electrode. Note that, to avoid sudden ignition, H_2_- and O_2_-containing gases were mixed in a bubbler bottle with water. (B) H_2_ and O_2_ concentrations in culture medium were monitored with specific electrodes. Under H_2_-mixed gas, the H_2_ concentration was maintained at 390±40 μM. Under both H_2_- and N_2_-mixed gases, the O_2_ concentration was maintained at 120±10 μM.

H_2_-mixed gas was composed of 10% O_2_, 5% CO_2_, 35% N_2_, and 50% H_2_ (purity > 99.999%; Iwatani, Tokyo, Japan). N_2_-mixed gas was composed of 10% O_2_, 5% CO_2_, and 85% N_2_. Cells grown on culture dishes (5 × 10^4^/cm^2^) for 1 day in a 5% CO_2_ incubator were set in acrylamide boxes (6.8 × 10^3^/cm^3^), which were strewn with wet paper to prevent desiccation. The boxes were sealed and filled with an appropriate mixed gas at a flow rate of 1 L/min for 30 min under normal pressure (Fig 1A). After filling, cells in the boxes were incubated at 37°C for the indicated duration. Immediately after incubation, the H_2_ and O_2_ concentrations in the culture medium were monitored with specific electrodes (Unisense, Aarhus N, Denmark). Under H_2_-mixed gas, the H_2_ concentration was maintained at 390±40 μM. Under both H_2_- and N_2_-mixed gases, the O_2_ concentration was maintained at 120±10 μM (Fig 1B).

### Cell survival assay

Cells (5 × 10^4^/cm^2^) grown in a 24- or 96-well plate for 1 day were used. For pretreatment with mixed gases, cells were incubated in a box containing an appropriate mixed gas for the indicated duration. Immediately after exposure to the mixed gas, the culture medium was replaced with DMEM containing 1% FBS with/without H_2_O_2_ and cells were further incubated in a conventional 5% CO_2_ incubator for 18 h. For post-treatment with mixed gases, the culture medium was replaced with DMEM containing 1% FBS with/without H_2_O_2_ and cells were further incubated in a box filled with an appropriate mixed gas for 18 h. After incubation, cell viability was estimated by a modified 3-(4,5-dimethylthiazol-2-yl)-2,5-diphenyl tetrasodium bromide viability assay according to the manufacturer’s instructions (WST-1 assay, DOJINDO, Kumamoto, Japan) or a two-color fluorescence cell viability assay (LIVE/DEAD Viability/Cytotoxicity assay, Thermo Fisher, Waltham, MA, USA), which is based on the simultaneous determination of intracellular esterase activity and plasma membrane integrity.

### Evaluation of mitochondrial membrane potential

To monitor (ΔΨ_m_, JC-1 dye (Thermo Fisher) was used. After incubation in a box containing an appropriate mixed gas, cells in a 24-well plate were stained with 20 μM JC-1 for 10 min and then evaluated by confocal microscopy. Red aggregates and green monomers were recorded with excitation (Ex) at 535 nm/emission (Em) at 590 nm and Ex at 485 nm/Em at 535 nm, respectively. Relative (ΔΨ_m_ was analyzed using Image J software (Version 1.44, NIH, Bethesda, MD, USA). Data are shown as the arbitrary aggregate fluorescence units in three separate experiments.

### Oxygen consumption rate

The oxygen consumption rate in state 3 was determined at 37°C using high-resolution respirometry (Oroboros Oxygraph-2k, Innsbruck, Austria), essentially according to the protocol provided by the manufacturer. The resting respiration rate (state 2, the absence of adenylates) was measured following the transfer of 5 × 10^6^ cells to glass chambers in 2 ml of mitochondrial respiration medium (0.5 mM EGTA, 3 mM MgCl_2_, 60 mM K-lactobionate, 20 mM taurine, 10 mM KH_2_PO_4_, 20 mM HEPES, 110 mM sucrose, and 1 g/l bovine serum albumin, pH 7.1). After measurement of endogenous respiration, 5 mM pyruvate, 10 mM glutamate, and 2 mM malate were added, and cells were permeabilized with 50 μg of digitonin. Complex I respiration was specifically assessed through the further addition of 1 mM ADP, followed by titration of 10 mM succinate as the complex II substrate to measure complex I and II respiration.

### ATP production

Cellular ATP in each 96-well culture plate was extracted and reacted with luciferin-luciferase according to the manufacturer’s instructions (Cellular ATP Measurement System, TOYO INK, Tokyo, Japan). Bioluminescence was measured with a microplate luminometer (Envision, PerkinElmer, MA, USA).

### Cellular oxidative stress

To monitor the accumulation of mitochondrial thiol, after incubation in a box containing an appropriate mixed gas, cells in a 24-well plate were stained with 1 μM MitoTracker Red (MTR, Thermo Fisher) for 10 min, fixed with 4% paraformaldehyde, washed twice with phosphate-buffered saline, and then evaluated by confocal microscopy with Ex at 535 nm/Em at 590 nm. Cells were imaged and analyzed using Image J software. Data are shown as the arbitrary fluorescence units in three separate experiments.

Total glutathione and oxidized glutathione (GSSG) were measured using a luminescence-based system (GSH/GSSG-Glo Assay, Promega, Madison, WI, USA). Cells were washed with Hank’s Balanced Salt Solution and lysed with lysis regents. The GSH/GSSG ratio was calculated directly from luminescence measurements (in relative light units, RLU) using the following formula: GSH/GSSG ratio = (total glutathione RLU—GSSG RLU) / (GSSG RLU/2).

To monitor mitochondrial superoxide and cellular ROS, cells were incubated with 5 μM MitoSOX Red (Thermo Fisher) and 1 μM 5-(and-6)-chloromethyl-2′,7′-dichlorodihydrofluorescein diacetate, acetyl ester (Thermo Fisher) prepared in Hank’s Balanced Salt Solution for 10 min at 37°C, washed three times, and then scanned using a microplate fluorometer (Envision) with Ex at 510 nm/Em at 580 nm and Ex at 495 nm/Em at 520 nm, respectively.

### Cell staining

Immunocytochemistry was performed as previously described [15]. Cells were fixed with 4% paraformaldehyde prepared in phosphate-buffered saline, permeabilized with 0.2% Triton X-100, and incubated with an anti-Nrf-2 rabbit polyclonal antibody (C-20, Santa Cruz, Dallas, TX, USA). After incubation with a BODIPY FL-conjugated secondary antibody (anti-rabbit IgG; Thermo Fisher), cells were visualized by confocal laser microscopy.

### Measurement of mitochondrial DNA copy number

Total DNA was isolated from cells using proteinase K digestion followed by standard phenol/chloroform extraction and ethanol precipitation, and subjected to TaqMan probe-based quantitative PCR analysis using a real-time PCR system (StepOnePlus, Thermo Fisher). The primers and probes for MT-ND1 gene in the mitochondrial DNA (mtDNA) and 18S rRNA gene in the nuclear DNA (nDNA,) used are listed in Table 1. The mtDNA copy number was calculated as a ratio of mtDNA/nDNA.

**Table 1:** Primers and probes used for semiquantitative PCR.

Gene	Primer/probe sequences
MT-ND1	Forward primer: CACCCAAGAACAGGGTTTGT Reverse primer: TGGCCATGGGTATGTTGTTAA Probe: 6FAM/TTACCGGGCTCTGCCATCT/TAMRA
18S rRNA	Forward primer: TAGAGGGACAAGTGGCGTTC Reverse primer: CGCTGAGCCAGTCAGTGT Probe: VIC/AGCAATAACAGGTCTGTGATG/TAMRA
ACTB	Forward primer: CCTTCTACAATGAGCTGCGT Reverse primer: TGGATAGCAACGTACATGGC Probe: 56-FAM/ATCTGGGTC/ZEN/ATCTTCTCGCGGTTG/3IABkFQ
CAT	Forward primer: GAGCACAGCATCCAATATTCTG Reverse primer: TCCTCATTCAGCACGTTCAC Probe: 56-FAM/TGCCCGCAC/ZEN/CTGAGTAACGTTATC/3IABkFQ
GCLC	Forward primer: CTCAGACATTGGATGGAGAGTAG Reverse primer: GAGCAGTACCACAAACACCA Probe: 56-FAM/TCGACCCAT/ZEN/GGAGGTGCAATTAACA/3IABkFQ
GPX1	Forward primer: CTTCCCGTGCAACCAGTT Reverse primer: TCTCGAAGAGCATGAAGTTGG Probe: 56-FAM/TCGTTCTTG/ZEN/GCGTTCTCCTGATGC/3IABkFQ
GSR	Forward primer: CGATCTATOAOGCACTTACCA Reverse primer: GCATTTCATCACACCCAAGTC Probe: 56-FAM/CCAACCACC/ZEN/TTTTCTTCCTTGTTAGCAC/3IABkFQ
HMOX1	Forward primer: AGGCAGAGGGTGATAGAAGAG Reverse primer: CTCTGGTCCTTGGTGTCAT Probe: 56-FAM/AGAGCTGGA/ZEN/TGTTGAGCAGGAACG/31ABkFQ
SOD2	Forward primer: GCTTGGTTTCAATAAGGAACGG Reverse primer: GCTCCCACACATCAATCCC Probe: 56-FAM/CCACTGCAA/ZEN/GGAACAACAGGCC/3IABkFQ

### Semiquantitative PCR analysis of the mRNA levels of anti-oxidative enzymes

Cellular total RNA was extracted using an RNeasy Plus Mini kit (QIAGEN, Valencia, CA, USA) and subjected to reverse transcription with a first-strand synthesis system (SuperScript II, Thermo Fisher). Samples of the resulting cDNA were subjected to TaqMan probe-based quantitative PCR analysis using a real-time PCR system. The primers and probes used are listed in Table 1. Relative gene expression was calculated using the standard curve method. The mRNA levels were normalized to that of ACTB (β-actin) gene.

### Western blot analysis

Cells were homogenized in RIPA buffer and centrifuged (15,000 *g* at 4°C for 20 min), and the supernatants were collected and stored at −80°C. Denatured proteins (10 μg in each lane) were separated on a 10% acrylamide gel and electrotransferred onto a polyvinylidene fluoride (PVDF) membrane. The PVDF membrane was blocked with skim milk and incubated at 4°C overnight with primary polyclonal rabbit antibodies against γ-glutamylcysteine synthetase heavy subunit (γ-GCSc; 1:200, H-338, Santa Cruz), heme oxygenase-1 (HO-1; 1:500, ADI-SPA-896, Enzo Life Sci., Farmingdale, NY, USA), superoxide dismutase 2 (SOD2; 1:200, FL-222, Santa Cruz) and polyclonal goat antibodies against NAD(P)H quinone oxidoreductase 1 (NQO1; 1:200, C-19, Santa Cruz), Nrf2 (1:200, T-19, Santa Cruz). After washing, membranes were incubated with peroxidase-conjugated goat anti-rabbit IgG (1:10000, Jackson ImmunoResearch, West Grove, PA, USA) or rabbit anti-goat IgG (1:10000, Jackson ImmunoResearch) at RT for 1 hour. Protein bands were detected using an enhanced chemiluminescence kit (ECL prime, GE Healthcare, Chicago, IL, USA) and visualized using an exposure and quantitation system (LAS-3000 mini, FUJI film, Tokyo, Japan). As a normalization control, the membranes were reprobed for 3-phosphate dehydrogenase (GAPDH) and exposed to polyclonal rabbit antibody against GAPDH (1:1000, Cell Signaling, Danvers, MA, USA).

### Statistical analysis

All experiments were repeated two or three times with similar results (n = 4–5 per group). Statistical analysis was performed by applying a one-way ANOVA with the Bonferroni correction or the Student’s *t*-test. Data are presented as mean ± SD. Results were considered significant at *P* < 0.05. A two-way ANOVA was used to evaluate effects of pretreatment with mixed gases for different durations on cell death; a significant interaction was interpreted by a subsequent simple-effects analysis with the Student’s *t*-test.

## Results

### Protective effect of H_2_ pretreatment against H_2_O_2_-induced cell death

**Fig 2:**
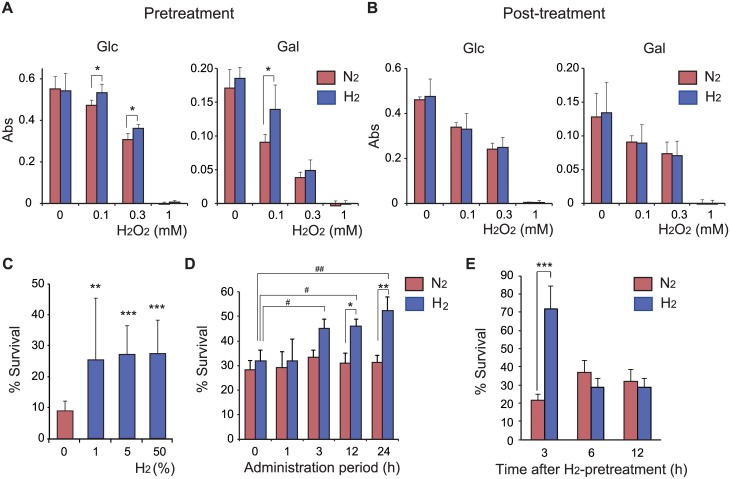
Protective effect of H_2_ pretreatment against H_2_O_2_-induced cell death. (A) For pretreatment with mixed gas, SH-SY5Y cells were incubated in culture medium containing either Glc or Gal under N_2_- or H_2_-mixed gas for 18 h. Immediately after the end of exposure to the mixed gas, the medium was replaced with fresh medium containing the indicated concentration of H_2_O_2_. Cells were further incubated in a conventional CO_2_ incubator for 18 h. (B) For post-treatment with mixed gases, culture medium containing either Glc or Gal was replaced with fresh medium containing the indicated concentration of H_2_O_2_. Cells were further incubated under an appropriate mixed gas. After the final incubation, cell viability was estimated by a modified 3-(4,5-dimethylthiazol-2-yl)-2,5-diphenyl tetrasodium bromide viability assay (A, B). (C) The protective effects of pretreatment with mixed gas containing different concentrations of H_2_ against 0.5 mM H_2_O_2_-induced cell death were examined. (D) The protective effects of pretreatment with N_2_- or H_2_-mixed gas for different durations against 0.5 mM H_2_O_2_-induced cell death were examined. Applying two-way ANOVA showed significant effects of mixed gas (*P* = 0.0017) and duration (*P* = 0.0315), however no interaction between them was observed (*P* = 0.2224). (E) After pretreatment with N_2_- or H_2_-mixed gas, culture medium was replaced with fresh medium, cells were incubated in a conventional CO_2_ incubator for the indicated duration, and then H_2_O_2_ (final 0.5mM) was added. Cells were further incubated for 18 h. All cells used in C—E were cultured in medium containing Glc. After the final incubation, cell viability was estimated by a two-color fluorescence cell viability assay and expressed as a percentage compared with cells not treated with H_2_O_2_ (considered as 100%) (C—E). *P < 0.05, **P < 0.01, ***P < 0.001 versus treatment with N_2_-mixed gas. ^#^*P* < 0.05, ^##^*P* < 0.01 versus an administration period of 0 h.

To investigate the molecular mechanisms underlying the physiological function of H_2_, we used human neuroblastoma SH-SY5Y cells because several lines of evidence indicate that treatment with H_2_ effectively protects against neuronal damage *in vivo* [3]. To prevent an accidental change in the O_2_ concentration, which would strongly affect cellular signaling and cell fate, we established a new cell culture system under strict gas control and carefully monitored the concentrations of H_2_ and O_2_ in the medium (Fig 1). SH-SY5Y cells were cultured in medium containing either Glc or Gal. Substituting Gal for Glc in cell culture media enhances mitochondrial metabolism [16]. Pretreatment of cells with 50% H_2_ gas for 18 h in both Glc- and Gal-containing media suppressed H_2_O_2_-induced cell death (Fig 2A), whereas post-treatment did not (Fig 2B). The protective effect of pretreatment was dose-dependent, and H_2_ concentrations of 1% and higher were significantly effective (Fig 2C). Pretreatment with 50% H_2_ for 3 h was sufficient to elicit the protective effect, whereas pretreatment for 1 h was not (Fig 2D). Cells were still protected against H_2_O_2_ toxicity at 3 h after the end of pretreatment with 50% H_2_, whereas after 6 h they were not (Fig 2E), indicating that the protective effects of pretreatment are transient in growing cultured cells.

### Enhancement of mitochondrial activity by H_2_ treatment

**Fig 3:**
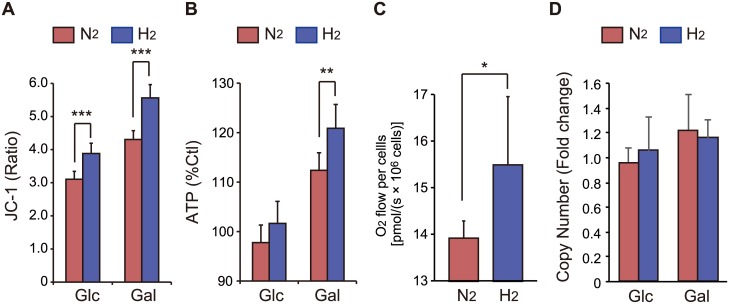
Enhancement of mitochondrial activities by H_2_ treatment. (A) H_2_ treatment enhanced JC-1-indicated ΔΨ_m_, which was expressed as the ratio of monomers to aggregates. (B) H_2_ treatment enhanced the accumulation of ATP, which was expressed as a percentage compared with cells not treated with mixed gases (considered as 100%). SH-SY5Y cells were incubated in culture medium containing either Glc or Gal under N_2_- or H_2_-mixed gas for 18 h. **P < 0.01, ***P < 0.001 (A, B). (C) H_2_ treatment enhanced the O_2_ consumption rate in state 3. O_2_ consumption was monitored with high-resolution respirometry. *P < 0.05. (D) The mtDNA copy number relative to nDNA in cells incubated under H_2_-mixed gas was quantified by real-time PCR analysis, and expressed relative to those in cells incubated under N_2_-mixed gas.

We previously observed that H_2_ prevents the antimycin A (a mitochondrial respiratory complex III inhibitor)-dependent decline in ΔΨ_m_ [4]. We used the ΔΨ_m_ indicator JC-1 to determine the physiological changes of mitochondria in intact cells treated with 50% H_2_ for 18 h. H_2_ treatment significantly increased ΔΨ_m_ in both Glc- and Gal-containing media (Fig 3A). Furthermore, H_2_ treatment increased the accumulation of cellular ATP (Fig 3B), indicating that it activates OXPHOS and then enhances mitochondrial energy production. To confirm the activation of mitochondrial OXPHOS, we measured cellular O_2_ consumption by high-resolution respirometry. H_2_ treatment enhanced the O_2_ consumption rate in state 3 (Fig 3C). On the other hand, the mtDNA copy number relative to nuclear genes was not affected by H_2_ treatment (Fig 3D), indicating that the increase in oxygen consumption per cell was not due to a change in the mitochondrial copy number.

### Induction of weak oxidative stress by H_2_ treatment

**Fig 4:**
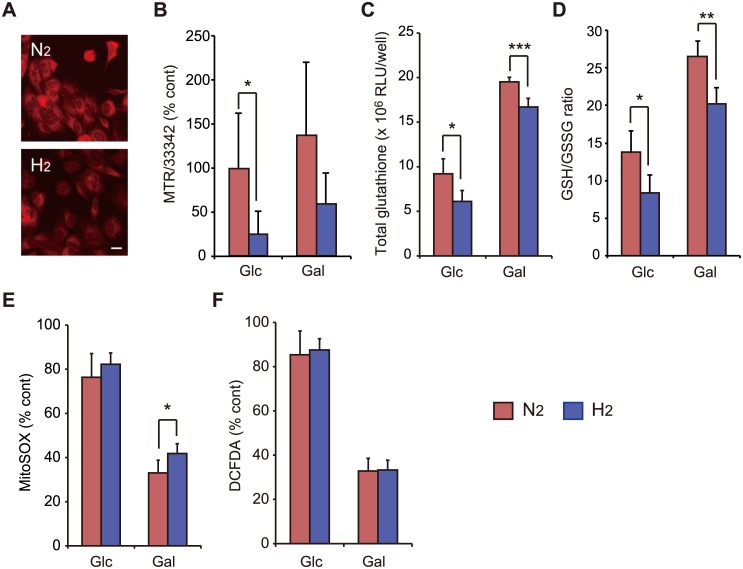
Induction of oxidative stress by H_2_ treatment. SH-SY5Y cells were incubated in culture medium containing either Glc or Gal under N_2_- or H_2_-mixed gas for 18 h. (A, B) H_2_ treatment attenuated staining with MTR, which was expressed as a percentage compared with cells not treated with mixed gases (considered as 100%). The scale bar is 20 μm in (A). (C, D) H_2_ treatment decreased the total glutathione and GSH levels. (E) H_2_ treatment increased superoxide formation in cells cultured in Gal-containing medium, which was expressed as a percentage compared with cells not treated with mixed gases (considered as 100%). (F) H_2_ treatment did not increase DCFDA-indicated ROS, which was expressed as a percentage compared with cells not treated with mixed gases (considered as 100%). *P < 0.05, **P < 0.01, ***P < 0.001.

To monitor the effects of mitochondrial hyperactivity induced by H_2_ treatment on oxidative stress, we first used MTR. This dye contains a mildly thiol-reactive chloromethyl moiety, which keeps it associated with mitochondria after fixation [17]. H_2_ treatment attenuated staining with MTR, indicating that it decreased the thiol concentration in mitochondria (Fig 4A and 4B). H_2_ treatment decreased the total glutathione and GSH levels (Fig 4C and 4D), indicating that the decrease in the thiol concentration was at least partially dependent on that in GSH. H_2_ treatment significantly increased superoxide formation in cells cultured in Gal-containing medium (Fig 4E), whereas it did not increase the formation of DCFDA-indicated ROS (Fig 4F). Both the decrease in GSH and the increase in superoxide strongly suggest that H_2_ treatment induces oxidative stress in cells. However, H_2_-induced oxidative stress was too weak to affect cell survival (Fig 2A and 2B).

### Induction of the anti-oxidative defense system by H_2_ treatment

**Fig 5:**
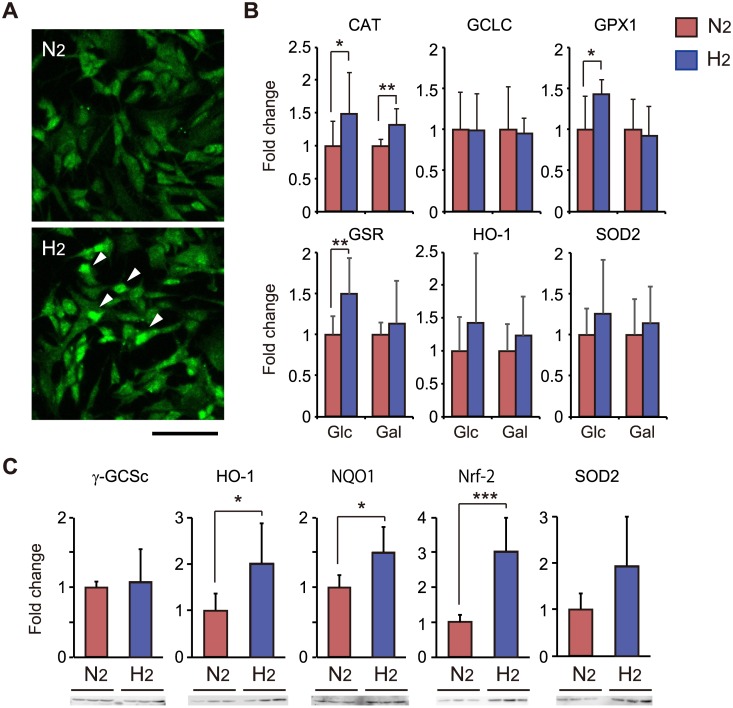
The H_2_-induced anti-oxidative defense system. (A) SH-SY5Y cells were stained with an anti-Nrf2 antibody. H_2_ treatment induced translocation of Nrf2 into the nucleus (arrow heads). The scale bar is 100 μm. (B) Cells were incubated in culture medium containing either Glc or Gal under N_2_- or H_2_-mixed gas for 18 h. The transcript levels of genes involved in the anti-oxidative defense system, including CAT, GCLC, GPX1, GSR, HO-1 and SOD2, were quantified by real-time PCR analysis coupled with reverse transcription of total RNA, and expressed relative to those in cells incubated under N_2_-mixed gas. (C) The expression levels of proteins involved in the anti-oxidative defense system, including γ-GCSc, HO-1, NQO1, Nrf2 and SOD2, were quantified by the intensity of representative immunoblots (n = 5 or 6, each), and expressed relative to those in cells incubated under N_2_-mixed gas. *P < 0.05, **P < 0.01. ***P < 0.001.

Weak oxidative stress is expected to be associated with triggering of an adaptive response to stronger oxidative stress [18]. To investigate induction of the anti-oxidative system, we stained cells with an anti-Nrf2 antibody. H_2_ treatment induced translocation of the transcription factor Nrf2 into the nucleus (Fig 5A). We further measured the expression of anti-oxidative enzymes underlying the Nrf2 pathway by performing quantitative PCR. After H_2_ treatment, transcription of CAT, GPX1 and GSR genes increased significantly (Fig 5B). Western blot of cell extracts also revealed that H_2_ treatment resulted in an increase in HO-1, NQO1 and Nrf2 expressions (Fig 5C), indicating that weak oxidative stress induced by H_2_ treatment evokes an anti-oxidative defense system in SH-SY5Y cells.

## Discussion

Increasing evidence suggests that some natural products and synthetic drugs that improve our health may act via a characteristic process called hormesis, in which adaptive responses against cellular stress are activated [19, 20]. In the current study, we found that H_2_ pretreatment effectively protects against H_2_O_2_-induced neuronal damage *in vitro*; H_2_-induced slight activation of mitochondria accompanied by weak oxidative stress triggers adaptive responses against oxidative stress.

　H_2_ selectively reduces the highly toxic ROS OH· and peroxynitrite, but not O_2_^-^·, H_2_O_2_, or nitric oxide [4]. The cellular toxicity of H_2_O_2_ is partially explained by the production of ·OH, which leads to alterations of lipids, proteins, and nucleic acids [21]. We previously observed that H_2_ reduces a large amount of ·OH produced by the Fenton reaction, irradiation, or sonication *in vitro* [4–6], strongly supporting the conclusion that a sufficient amount of H_2_ can efficiently moderate cellular oxidative damage induced by ·OH. However, treatment with 50% H_2_ gas after the addition of H_2_O_2_ (post-treatment) did not protect cells (Fig 2A and 2B). Because of the relatively low reactivity of H_2_ against ·OH [22], we speculate that the steady-state concentration of ·OH derived from exogenous H_2_O_2_ in the cell is too low to react with H_2_. On the other hand, pretreatment with H_2_ ameliorated H_2_O_2_-induced cell death. When H_2_O_2_ was added, no detectable amount of H_2_ remained in the cell (data not shown), suggesting that the pretreatment dose of H_2_ did not directly prevent H_2_O_2_-induced oxidative damage, but provoked an anti-oxidative defense system against H_2_O_2_ in our experiments.

The protective effect of H_2_ pretreatment was dose-dependent, and H_2_ concentrations of 1% and higher were significantly effective (Fig 2C). Inhalation of H_2_ gas is the simplest method to intake H_2_ and ameliorates acute diseases such as ischemia-reperfusion and graft injuries of several organs [1, 2]. The effective concentration of H_2_ in mixed gas is usually maintained between 1% and 4% [4, 23, 24]. On the other hand, drinking water saturated with H_2_ (1.6 ppm, saturated with 100% H_2_ gas) is safer and more convenient than inhaling H_2_ gas. After administration of HW containing a saturating amount of H_2_ (80%) with a feeding needle in mouse, the H_2_ concentration in the liver is immediately elevated, peaking at approximately 2% H_2_ [25]. These observations indicate that about a few percent of H_2_ is the physiologically active concentration *in vivo*.

H_2_ treatment significantly increased ΔΨ_m_ and the cellular ATP level, indicating that H_2_ treatment enhances mitochondrial energy production via activation of OXPHOS (Fig 3). This was supported by the observation that H_2_ treatment enhanced the O_2_ consumption rate. OXPHOS is regulated through respiratory control, allosteric regulation, and post-translational modifications [26]. Calcium is the strongest signal for mitochondrial activation [27]. Although the direct target molecule of H_2_ in physiological conditions remains to be identified, several potential effectors of H_2_ have been discussed, including cell signaling molecules and hormones that are responsible for preventing oxidative stress and inflammation. Recently, Iuchi *et al*. demonstrated that low concentrations (approximately 1%) of H_2_ modulate Ca^2+^ signal transduction [28], indicating that excessive calcium signaling enhances OXPHOS activity.

OXPHOS in a hyperactive state leads to hyperpolarization of ΔΨ_m_, which causes the generation of excessive ROS [29]. H_2_ treatment attenuated staining with MTR after fixation (Fig 4A). Because the chloromethyl moiety of MTR is thiol-reactive, we speculated that H_2_ treatment decreases the thiol concentration in mitochondria. We then measured the intracellular concentration of GSH, which is the most abundant thiol inside the cell [30]. H_2_ treatment decreased the total amount of glutathione, concomitant with a lower GSH/GSSG ratio (Fig 4C and 4D). Finally, treatment with the selective reductant H_2_ enhanced the accumulation of the mitochondrial ROS O_2_^-^· (Fig 4E), indicating that H_2_ induces weak oxidative stress.

Increasing evidence indicates that transient exposure to low levels of ROS can protect neurons against subsequent exposure to a lethal level of oxidative stress [18]. The Keap1/Nrf2/ARE pathway is one of the molecular mechanisms underlying this adaptive response. Indeed, H_2_ treatment induced the translocation of Nrf2 into the nucleus and enhanced the transcription of CAT, GPX1 and GSR and the accumulation of HO-1, NQO1 and Nrf-2 (Fig 5). H_2_ pretreatment for more than 3 h was required to protect cells (Fig 2D). We speculated that exposure for 1 h was too short to induce oxidative stress sufficient to provoke an anti-oxidative defense system via transcriptional regulation.

In our experimental condition, a high dose of H_2_O_2_ was used to induce cell death, accompanied by a decrease in mitochondrial activity, which was not prevented by the post-treatment with H_2_ (Fig 2B). On the other hand, the post-treatment with H_2_ did not enhance H_2_O_2_-dependent cell death, indicating that H_2_-induced oxidative stress is negligible and very weaker than that induced by H_2_O_2_ during cell death. It was recently found that a low dose of H_2_O_2_ also plays a crucial role in the induction of hormesis [31], suggesting that the simultaneous treatment with H_2_ and a low dose of H_2_O_2_ may coordinately enhance a defense system against oxidative stress. Further studies will be required to elucidate the hormesis effect of H_2_ under weak oxidative stress.

With or without post-treatment with 0.3 mM H_2_O_2_ induce cell death. However, treatment with 50% H_2_ gas after the addition of H_2_O_2_ (post-treatment) did not protect cells (Fig 2A and 2B). Because of the relatively low reactivity of H_2_ against ·OH [22], we speculate that the steady-state concentration of ·OH derived from exogenous H_2_O_2_ in the cell is too low to react with H_2_.

After drinking of HW, hypoxia-reoxygenation-induced superoxide formation in a mouse brain slice is suppressed [32]. Noteworthy, there is no trace amount of H_2_ in the slice, indicating that HW induces anti-oxidation systems in the brain. We also found that preadministration of HW to mice before lipopolysaccharide injection prolongs survival and reduces oxidative stress in the liver, with increased expression of HO-1 and decreased expression of ET-1 [33].

In summary, H_2_ pretreatment prevented H_2_O_2_-induced cell death, enhanced mitochondrial activities, accompanied by an increased level of oxidative stress, and then induced expression of anti-oxidative enzymes. Based on H_2_-induced adaptive responses *in vitro* and *in vivo*, we now consider that H_2_ functions as a so-called mitohormetic effector against oxidative stress.
